# Prognostic significance of Versican expression in gastric adenocarcinoma

**DOI:** 10.1038/oncsis.2015.36

**Published:** 2015-11-30

**Authors:** X-H Shen, W-R Lin, M-D Xu, P Qi, L Dong, Q-Y Zhang, S-J Ni, W-W Weng, C Tan, D Huang, Y-Q Ma, W Zhang, W-Q Sheng, Y-Q Wang, X Du

**Affiliations:** 1 Department of Pathology, Fudan University Shanghai Cancer Center, Shanghai, China; 2 Clinical and Pathological Diagnosis Center, Ningbo, China; 3 Institute of Pathology, Fudan University, Shanghai, China; 4 Institute of Biomedical Sciences, Fudan University, Shanghai, China; 5 Department of Pathology, First Hospital Affiliated to Xinjiang Medical University, Wulumuqi, China; 6 Department of Pathology, Obstetrics and Gynecology Hospital of Fudan University, Shanghai, China

## Abstract

Gastric cancer (GC) is the leading malignancy in the digestive system. Versican is a ubiquitous component of the extracellular matrix and has a role in tumor progression. We aim to examine the expression of Versican in GC and the relationship between Versican levels and patient survival. We detected the mRNA expression of Versican in tumorous pairs and adjacent normal tissues (ANTs) of 78 GC patients by quantitative real-time polymerase chain reaction. The protein expression of Versican in 101 cases of matched GC and ANT, as well as in 27 intraepithelial neoplastic (IN) samples, was evaluated by immunohistochemistry. We analyzed the correlation between Versican levels and clinical outcomes. Finally, we performed CCK-8 cell counting assay and transwell assay in GC cell lines. Versican mRNA expression was significantly greater in tumor tissues (*P*<0.001) than in ANT. Versican was majorly expressed in the stroma surrounding tumor epithelium and minorly some areas of tumor epithelium. The Versican expression level was higher in GC than in ANT (*P*=0.004), but no significant difference was observed between ANT and IN (*P*=0.517). The Versican mRNA and protein levels were consistent in GC. High Versican mRNA and protein expression correlated with greater tumor invasion depth (*P*=0.030, *P*=0.027). Univariate and multivariate analysis revealed that patients with high Versican mRNA expression exhibited poor disease-specific survival (*P*<0.001). *In vitro* experiments showed that Versican overexpression promoted cell proliferation and invasion. Our data indicate that Versican may be a novel prognostic indicator in GC and may be a potential target for clinical diagnosis.

## Introduction

Gastric cancer (GC) remains one of the most common malignancies and most frequent cause of cancer death.^1, 2^ The majority of GC is gastric adenocarcinoma, which has a relatively high 5-year survival rate despite of the early-stage diagnosis.^1^ Clinical prognostic factors such as histological type and stage supply limited predictive information for the subsequent treatment of gastric adenocarcinoma, and new biological markers are in demand to achieve the most effective diagnostic method.

The extracellular matrix (ECM), composed of proteoglycans (PGs), glycoproteins and collagens, is a highly organized structure with many physiological and pathological roles.^3^ Modifying the ECM composition through a large array of molecules, as well cell–cell and cell–matrix interactions, may be crucial for tumor initiation and progression.^4^ PGs, a group of heavily glycosylated proteins, are present throughout the mammalian body and are involved in a wide variety of biological phenomena, including structural maintenance, tissue remodeling, molecular presentation, cellular adhesion and signal transmission. Using intracellular signaling pathways, PG and growth factor signals are transmitted and alter the cellular response,^5^ thus, PGs are directly implicated in a large variety of human diseases. Several studies indicated that significant PG content changes occur in the tumor stroma of epithelial neoplasms, thereby supporting tumor growth and invasion. Versican, a member of the aggregating chondroitin sulfate PGs family,^6^ is accumulated predominantly in the tumor stroma,^7^ providing hygroscopic properties to create a loose and hydrated matrix that is necessary to support key events in development and disease. Through direct or indirect interactions with cells and molecules, Versican has significant roles in modulating cell proliferation, differentiation, adhesion and migration, all of which are features of cancer invasion and metastasis.^8^ Thus, Versican may serve a wide range of functions in the invasion and metastasis of tumor cells.

Previous studies have shown that Versican can be detected in many malignancies including melanoma, non-small cell lung cancers, breast tumors,^9, 10, 11, 12^ pharyngeal,^13^ ovarian cancer^7, 14^ and cervical cancer.^15^ Increased accumulation of structurally modified Versican is reportedly related to the progression of laryngeal cancer.^16^ However, the significance of Versican has been less extensively studied in gastric adenocarcinoma. The aim of our study was to investigate Versican expression in gastric adenocarcinoma and to determine its relationship with clinicopathologic factors, with a special emphasis on its prognostic significance.

## Results

### Versican expression in tissues

**Figure 1:**
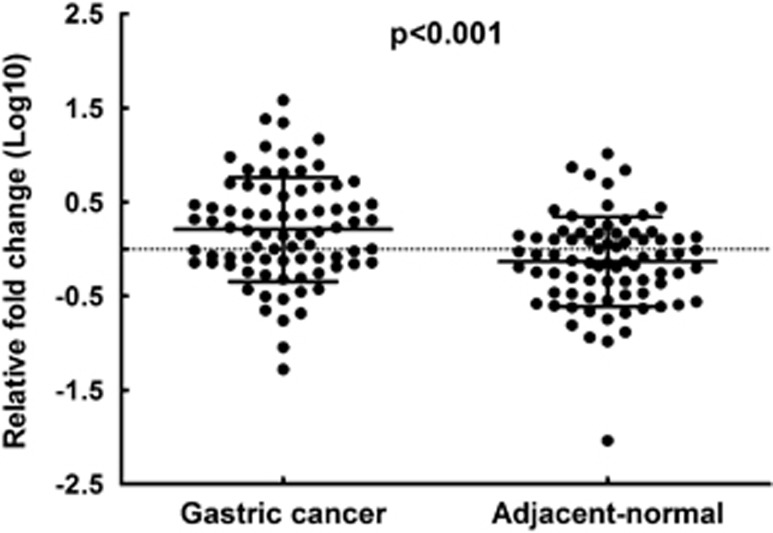
Versican expression as assessed by quantitative RT polymerase chain reaction (qRT–PCR) in cancerous tissue (GC) and adjacent normal mucosa (ANT). Versican expression was significantly higher in cancerous tissues compared with ANTs. Data represent means±s.d. (*P*<0.001). Beta-actin was used as an endogenous control to normalize the data.

The first goal of our study was to investigate whether Versican is detectable and altered in gastric adenocarcinoma tissues compared with adjacent normal ones. So we performed quantitative real-time (RT) polymerase chain reaction with RNAs isolated from gastric adenocarcinoma tissues to detect the expression levels of Versican. It turned out that Versican expression was significantly greater in tumor tissues compared with adjacent normal tissues (ANTs; *P*<0.001; Figure 1), suggesting that Versican was highly expressed in malignant gastric adenocarcinoma.

### Versican expression in GC tissues, IN and ANTs

**Figure 2:**
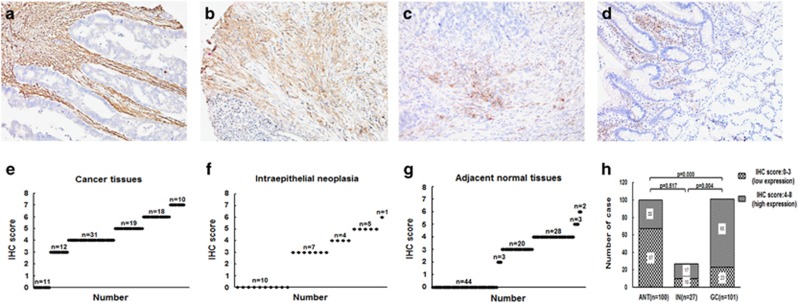
Versican expression as assessed by immunohistochemistry in cancerous tissue, IN and adjacent normal mucosa. Representative immunostaining results of Versican in **(a)** GC (moderately differentiated), intense stromal Versican staining; **(b)** GC (poorly differentiated), moderate stromal Versican staining; (**c**) GC (poorly differentiated), mild stromal Versican staining; **(d)** IN, mild stromal Versican staining (x200 magnification). IHC score of stromal Versican expression in **(e)** GC, **(f)** IN and **(g)** ANT. **(h)** Scoring differences between GC and ANT, GC and IN, and ANT and IN (*P*=0.000, *P*=0.004 and *P*=0.517, respectively).

Next, we performed immunohistochemistry to examine the protein expression of Versican in 101 GC tissue samples, 100 ANT samples (one sample missing) and 27 intraepithelial neoplastic (IN) samples. First, we observed that the staining of Versican was in nucleus or both nucleus and cytoplasm. The staining areas majorly covered the stromal cells characteristically surrounding the epithelial lesions, and also covered some part of the epithelium of cancer areas (Figures 2a–d). The staining of Versican was significantly more intense in gastric adenocarcinoma cases than that in either IN or ANT tissues (Figures 2a–d). When scored using the Ruiter *et al.* scoring system,^13, 17^ more cases with high Versican expression were observed in gastric adenocarcinoma (78 out of 101, 77.23% Figure 2e). Compared with IN (17 out of 27, 62.96% Figure 2f) and ANT (33 out of 100, 33.00% Figure 2g), the proportion of high Versican expression was significantly larger in gastric adenocarcinoma than that in either IN (*P*=0.004) or ANT (*P*=0.000; Figure 2h).

**Figure 3:**
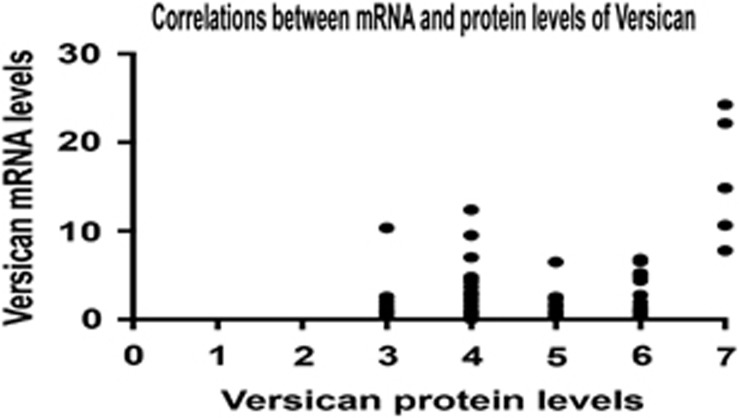
The correlation between mRNA and protein levels of Versican in gastric adenocarcinoma. A Pearson correlation analysis was done between the mRNA levels of and protein levels of Versican in 78 patients with gastric adenocarcinoma. (*R*=0.344, *P*<0.01).

Furthermore, we chose 50 cases with both results of RT–quantitative polymerase chain reaction (qPCR) in tumor tissues and immunochemistry in paraffin sections to examine the correlation between mRNA level and protein expression of Versican. As shown in figure, the mRNA levels of Versican in these 50 cases were highly correlated with protein expression levels in immunochemistry (Figure 3). Taken together, these data suggested that Versican was highly expressed in gastric adenocarcinoma both on mRNA and protein levels.

### Versican expression and clinicopathological factors of GC

To assess the correlation between the Versican expression and clinicopathological data, the Versican expression in tumor tissues were categorized as low or high by the median value.^18^ At both the mRNA and protein levels, elevated Versican expression was correlated with greater tumor invasion than lower expression (*P*=0.030 and *P*=0.027, respectively). There was no significant correlation between Versican expression and other clinicopathologic features, such as age, gender, tumor size, tumor location, histologic grade, lymphatic metastasis, peritoneal metastasis, vascular invasion, nervous invasion or tumor stage (*P*>0.05; Table 1).

### Correlation between Versican expression and GC patient prognosis

**Figure 4:**
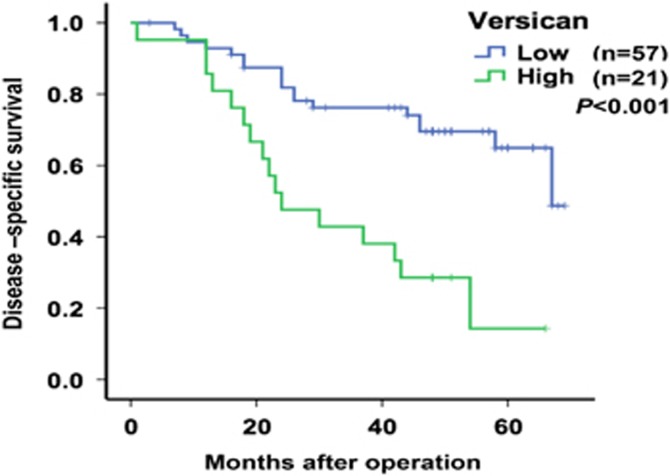
Kaplan–Meier survival curves of patients with different expression levels of Versican in GC. Patients with high Versican expression levels (*n*=57) exhibited significantly poorer prognoses than those with low Versican expression levels (*n*=21, *P*=0.000). *P*-values were calculated using the log-rank test.

Disease-specific survival (DSS) curves were plotted according to Versican mRNA expression levels using the Kaplan–Meier method. During follow-up, 20 patients died of gastric adenocarcinoma. As presented in Figure 4, the patients with high Versican expression exhibited a significantly poorer prognosis than those with low Versican expression (*P*<0.001). Univariate analysis of DSS revealed that the relative level of Versican expression (*P*<0.001; Figure 4), tumor depth (*P*=0.020), nervous invasion (*P*=0.024), lymphatic metastasis (*P*=0.004), peritoneal metastasis (*P*=0.027) and tumor stage (*P*<0.001) were prognostic indicators (Table 2). The other clinicopathological features, such as age, gender, tumor size, tumor location, histologic grade and vascular invasion, were not statistically significant prognosis factors (*P*>0.05; Table 2). Multivariate analysis revealed that in addition to the tumor stage (*P*=0.001), Versican expression was an independent prognostic indicator for DSS in GC patients (*P*=0.002; Table 2).

### Overexpression of Versican promotes tumor cell proliferation and invasion in gastric carcinoma

**Figure 5:**
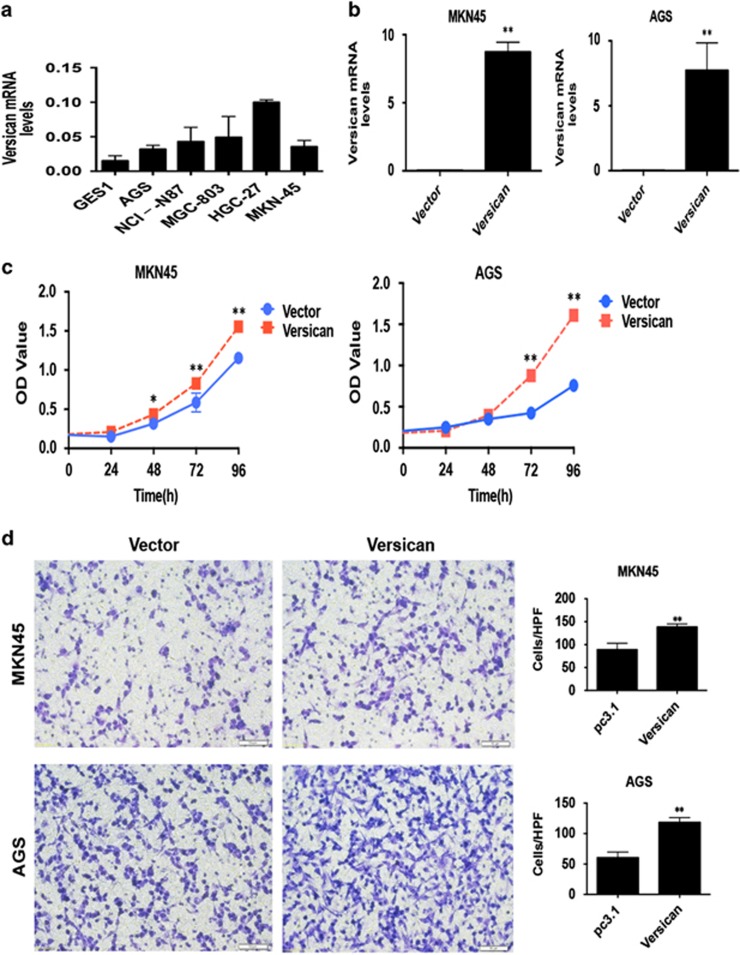
Overexpression of Versican promoted tumor cell proliferation and invasion in GC cell lines. (**a**) The mRNA levels of Versican were detected by RT–qPCR in GES1 and five GC cell lines. (**b**) The efficiencies of Versican in MKN45 and AGS cells were detected by RT–qPCR. (**c**) CCK-8 assay was performed to detect the influence of overexpression of Versican on tumor cell proliferation in MKN45 and AGS cells. *P<0.05; **P<0.01. (**d**) Transwell assay was performed to detected the influence of overexpression of Versican on tumor cell invasion in MKN45 and AGS cells. **P<0.01.

Finally, we turned to verify the biological functions of Versican *in vitro.* We detected the mRNA level of Versican in normal GES1 and five gastric carcinoma cell lines by RT–qPCR first (Figure 5a), and chose MKN45 and AGS as the candidate cell lines for interference (Figure 5b). Then, we used the Cell Counting Kit-8 (CCK-8) assay to examine the potential influence of Versican on cell proliferation in GC cell lines. As shown in figure, overexpression of Versican stimulated cell proliferation in MKN45 and AGS cells (Figure 5c). Next, we performed the Transwell assay to investigate the effect of Versican on cell invasiveness. Similarly, overexpression of Versican enhanced cell invasion in MKN45 and AGS cells (Figure 5d). These data together suggested that Versican promoted tumor cell proliferation and invasion in gastric carcinoma.

## Discussion

As far as it is concerned, our study is the first to report data for the prospective power of Versican expression in clinical cohorts. We found that Versican expression is associated with the clinicopathological features and prognosis of GC. We speculate that dysregulated Versican expression may contribute to GC development and/or progression, as well as to the prognosis of GC patients. Thus, Versican may be applied as a biomarker for malignancy and monitoring prognosis in gastric adenocarcinoma clinically.

Versican has been observed in many malignancies, including melanoma, epithelial ovarian cancer, breast cancer and non-small cell lung cancer,^7, 9, 10, 11, 15^ implying its importance in tumor maintenance. Stylianou *et al.*^16^ reported a 140-fold increase in Versican expression in laryngeal cancer tissue relative to normal controls. In serous ovarian cancer, Versican was reportedly expressed at significantly greater levels in tumor samples versus normal ovarian tissues.^14^ In our study, we first investigated altered Versican expression in 78 pairs of GC and ANT samples by RT–qPCR and confirmed that Versican expression was significantly greater in 78 GC tumor tissues relative to the respective ANT samples. Together with our studies of gastric adenocarcinoma, these data support the view that Versican mRNA expression levels may be associated with cancer development.

In different cancers, Versican deposits have been demonstrated both in tumor stroma^19, 20^ and in tumor cells.^12^ Analyses of mRNA have also demonstrated that tumor-associated Versican is synthesized both in the peritumoral stroma and in the tumor itself.^21^ Our study suggested that Versican expression was only detected in the tumor stroma surrounding epithelial lesions in gastric adenocarcinoma without any supplementary intracellular Versican accumulation, thereby suggesting that in GC, Versican is likely predominantly synthesized in the tumor stroma by stromal fibroblasts. The effects of stromal Versican expression on tumor progression may be dependent on the organ and tumor type examined.

Previous studies concerning Versican immunohistochemical staining have revealed that stromal Versican expression was significantly greater in the patients with advanced disease.^7^ Our data detected statistically significant differences in Versican expression levels between gastric adenocarcinoma and ANT, as well as between gastric adenocarcinoma and IN. The group with higher Versican mRNA and protein expression exhibited deeper tumor invasion than the lower Versican expression group. Moreover, we observed that patients with high Versican expression exhibited poor DSS, and multivariate analysis revealed that Versican expression was an independent prognostic indicator for DSS. Versican may be an applicable prognostic predictor and monitor for gastric adenocarcinoma in clinical practice. Future studies may testify its value in prognostic prediction in a larger sample of gastric adenocarcinoma or a sample containing multiple histological subtypes of gastric malignancies.

Neoplastic remodeling of the ECM by modulating stromal cell secretion of macromolecules, such as Versican, is a well-recognized phenomenon and may be one mechanism by which tumor cells control their microenvironment to facilitate local invasion and metastasis.^12, 13, 16, 22, 23^ Considering the correlation of high Versican expression with tumor metastasis and recurrence found in our study, Versican may promote tumor progression by prompting tumor invasion. We further identified that Versican was critical for enhancement of cancer cell proliferation as well as tumor cell invasion *in vitro.* As a ubiquitous component of the ECM, Versican is expressed and secreted by fibroblasts present in the tumor stroma and is likely regulated by transforming growth factor beta^17, 24, 25, 26^ and is also a target gene of Wnt signaling.^27^ The interplay of these factors may lead to different Versican expression levels in various tumor types. Indeed, Zhang *et al.*^28^ have suggested that Versican may exhibits biological effects in GC development by its two major functional isoforms V0 and V1, after triggered by an important cytokine interleukin-11, both isoforms participate in GC cells migration. The wide ranging functions of Versican have been attributed to its central glycosaminoglycan binding region, and to the N-(G1) and C-(G3)terminal globular domains, which collectively interact with a large number of ECM and cell surface structural components.^8^ Nevertheless, it is likely that target genes differ between specific tissues and cell types, and the specific target genes controlled by Versican in GC remain unknown. In future work, high-throughput techniques such as RNA sequencing and microarray analysis should be applied to obtain a global view of the changes of downstream molecules, thereby elucidating the regulation mechanisms of Versican.

### Conclusion

Our results show that Versican expression is significantly increased in GC tissues. Greater Versican expression levels were detected in more invasive tumors. In addition, the upregulation of Versican expression was associated with poor GC prognosis. These findings suggested that Versican may be a novel prognostic indicator in GC and may be a specific and accessible biomarker as well as a potential new target for GC clinical diagnosis.

## Materials and methods

### Tissue samples and clinical data collection

This study collected all 78 tissues of patients with gastric adenocarcinoma stored in tissue bank of Shanghai Cancer Center from 2009 to 2014. All the patients analyzed in this study underwent resection of primary gastric adenocarcinoma at Fudan University Shanghai Cancer Center from 2009 to 2014. All the cases were histopathologically confirmed as gastric adenocarcinoma according to WHO classifications of Tumors of Digestive System 2010 version. None of the patients underwent pre-operative treatment. All the resected tissue samples were immediately frozen in liquid nitrogen and stored at −80 °C until RNA extraction in the tissue bank. The data collected from all subjects included age, gender, DSS and clinicopathological features (for example, tumor size, location, histologic stage, depth of invasion, status of venous invasion, nervous invasion, lymphatic metastasis and peritoneal metastasis). The clinical stage was evaluated using the TNM classification system.^29^ Patient follow-up was performed every 2–3 months during the first postoperative year and 3–6 months thereafter, until 30 July 2015. All patients completed the follow-up. The DSS was defined as the length of time between the surgery and death from the cancer. During the follow-up, 20 patients died of disease. This study was approved by the research ethics committee of Fudan University Shanghai Cancer Center, and the participants provided informed consent for the use of their tissues in this study.

### RNA preparation, reverse transcription and quantitative RT polymerase chain reaction

Total RNA was extracted from the tumorous and ANTs using Trizol (Invitrogen, Carlsbad, CA, USA) following the manufacturer's protocol. RT and qPCR kits were used to evaluate the Versican expression from tissue samples. The RT and qPCR reactions were performed as previously described.^20^ Relative gene expression was calculated using the comparative cycle threshold (CT) (2^−ΔΔCT^) method, with beta-actin as the endogenous control to normalize the data.^30^ The following primers were used in this study: Versican, 5′-GATGTGTATTGTTATGTGGATCA-3′ (forward) and 5′-CATCAAATCTGCTATCAGGG-3′ (reverse); and beta-actin, 5′-TCCTCTCCCAAGTCCACACA-3′ (forward) and 5′-GCACGAAGGCTCATCATTCA-3′ (reverse).

### Immunohistochemistry and evaluation of immunohistochemical staining

A total of 101 GC sample pairs and 27 IN samples were studied. Slides and paraffin blocks were selected from the archives of the Pathology Department of Fudan University Shanghai Cancer Center. Immunohistochemical analysis was performed using 4-μm paraffin sections mounted on aminopropyl-triethoxysilane-coated slides. Sections were deparaffinized, rehydrated and treated with 0.3% H_2_O_2_ in methanol for 20 min to block endogenous peroxidase activity. Antigen retrieval was performed (0.01 m citrate, pH 6.0), and the sections were rinsed in phosphate-buffered saline. Subsequently, the sections were stained for Versican (overnight) using a 1:200 dilution of rabbit polyclonal anti-human Versican (ab111072, Abcam, Hong Kong, China) in phosphate-buffered saline containing 1% bovine serum albumin. Subsequently, the slides were incubated with Powervision-Poly-HRP-goat anti-mouse/rat/rabbit IgG (Gene Tech, Shanghai, China), and immune complexes were visualized with diamino-benzidine.

The expression of Versican was scored by two pathologists independently of the study. Only staining for nucleus (regardless of cytoplasmic staining) was regarded as positive. The expression was scored according to previous studies, which divided the staining intensity into none (0), mild (1), moderate (2) and intense (3) at low magnification (x200) and scored the percentage of positive tumor cells as 0% (0, absent), 1–5% (1, sporadic), 6–25% (2, local), 26–50% (3, occasional), 51–75% (4, majority) and 76–100% (5, large majority).^22, 31^ The combined scores were then divided into two groups by the middle value: category 0 (score 0–3, low expression) and category 1 (score 4–8, high expression). Human lung carcinoma tissue and normal gastric tissue are the positive and negative controls, respectively.^31^

### Plasmids

The full-length versican sequence was amplified by PCR from complementary DNA of NIH: AGS cells and then subcloned into pcDNA3.1 (+) vector (Transheep, Shanghai, China).

### Cell lines and culture conditions

Cell lines involved including human GES cell line, GC cell lines AGS, HGC-27, MKN-45, MGC-803, and NCI-N87 were cultured in Dulbecco's modified Eagle's medium (Gibco, Carlsbad, CA, USA) or RMPI-1640 (Gibco) respectively, supplemented with 10% fetal bovine serum (Gibco), 50 U/ml penicillin and 50 μg/ml streptomycin (Gibco). All cell lines were maintained at 37 °C, 5% CO_2_ in a humidified atmosphere.

### Cell proliferation assays

Cell proliferation was evaluated using CCK-8 (Dojindo, Kumamoto, Japan). In all, 2 × 10^3^ cells per well were seeded onto 96-well plated in 100 μl complete culture medium. The cells were cultured for 0, 24, 48, 72, 96 h before adding 10 μl CCK-8 (5 mg/ml) into the culture medium in each well. After a 2-h incubation at 37 °C, optical density at 450 nm was measured with an automatic microplate reader (Synergy4; BioTek, Winooski, VT, USA).

### Cell invasion assays

The transwell chambers (8 μm, 24-well format; Corning Co., New York, NY, USA) were used for cell invasion assay. A total of 4 × 10^4^ cells in 100 μl serum-free medium were loaded into the upper inserts, and 500 μl culture medium containing 10% fetal bovine serum were loaded into the lower chamber as chemo-attractant. After a 24-h incubation at 37 °C, the cells that had migrated through the filters were fixed with ethanol and stained with 0.1% crystal violet. Photographs were taken at 200 × under microscope (BX51, Olympus, Tokyo, Japan) and the number of invaded cells was counted at 400 × .

### Statistical analysis

All statistical analyses were performed using SPSS 20.0 software (IBM, SPSS, Chicago, IL, USA). The significance of between-group differences was estimated using Student's *t*-test, χ^2^ test or Wilcoxon test, as appropriate. DSS rates were calculated using the Kaplan–Meier method, with the log-rank test applied for comparison. Variables with a value of *P*<0.05 in univariate analysis were used in a subsequent multivariate analysis, based on the Cox proportional hazards model. Two-sided *P*-values were calculated, and a probability level of 0.05 indicated statistical significance. Statistical analyses of immunohistochemistry staining evaluation were performed using the Kruskal–Wallis one-way analysis of variance test and Mann–Whitney *U*-test.

## Figures and Tables

**Table 1:** Relationship between Versican expression and clinicopathologic parameters of gastric cancer patients

*Variables*	*mRNA expression*			*Protein expression*		
	*Low (*n*=57)*	*High (*n*=21)*	P-*value*^a^	*Low (*n*=31)*	*High (*n*=70)*	P-*value*^a^
*Age (years)*						
<60	25	11		17	34	
⩾60	32	10	0.339	14	36	0.358
						
*Gender*						
Male	44	18		25	55	
Female	13	3	0.314	6	15	0.520
						
*Tumor mass size*						
<5 cm	37	15		17	50	
⩾5 cm	20	6	0.399	14	20	0.082
						
*Location*						
Upper stomach	12	5		7	12	
Middle stomach	16	5		9	22	
Lower stomach	22	9		11	25	
Entire stomach	7	2	0.955	4	11	0.921
						
*Histologic grade*						
Well and moderately	5	15		21	53	
Poorly and others	16	42	0.536	10	17	0.274
						
*Depth of tumor*						
T1 and T2	15	1		2	17	
T3 and T4	42	20	0.030	29	53	0.027
						
*Vascular invasion*						
Absent	25	9		7	14	
Present	32	12	0.573	24	56	0.480
						
*Nervous invasion*						
Absent	27	6		22	55	
Present	30	15	0.108	9	15	0.279
						
*Lymphatic metastasis*						
Absent	19	6		9	25	
Present	38	15	0.456	22	45	0.338
						
*Peritoneal metastasis*						
Absent	47	16		13	29	
Present	10	5	0.372	18	41	0.566
						
*TNM stage*^b^						
I and II	23	6		13	31	
III and IV	34	15	0.247	18	39	0.501

a All statistical tests were two-sided. Significance level: *P*<0.05.

b Tumor stage was obtained according to the TNM criteria.

**Table 2:** Univariate and multivariate analysis of clinicopathological factors for disease special survival in gastric cancer

*Variable*	*Univariate analysis*		*Multivariate analysis*	
	*HR (95% CI)*	P*-value*^a^	*HR (95% CI)*	P-*value*^a^
Age (<60/⩾60)	0.676 (0.351–1.304)	0.243		
Gender (male/female)	0.991(0.432–2.269)	0.982		
Tumor size (<5/⩾5)	1.678 (0.863–3.261)	0.127		
Tumor location (upper/middle/lower/diffuse)	1.015 (0.715–1.442)	0.932		
Histologic grade (well, mod/poor, others)	1.075 (0.503–2.296)	0.852		
Depth of tumor (T1, T2/T3, T4)	5.936(1.424–24.751)	0.014	1.599 (0.298–8.576)	0.584
Vascular invasion (absent/present)	1.835 (0.916–3.678)	0.087		
Nervous invasion (absent/present)	2.605 (1.223–5.548)	0.013	1.198 (0.498–2.887)	0.687
Lymphatic metastasis (absent/present)	3.924 (1.521–10.124)	0.005	1.036 (0.133–8.0956)	0.973
TNM stage (I+II/III+IV)	5.567 (2.149–14.418)	0.000	7.711 (2.322–25.609)	0.001
Versican (low/high)	3.739 (1.933–7.232)	0.000	3.089 (1.533–6.226)	0.002

Abbreviations: CI, confidence interval; HR, hazard ratio.

a All statistical tests were two-sided. Significance level: *P*<0.05.
